# Designing a Ring-VCO for RFID Transponders in 0.18 **μ**m CMOS Process

**DOI:** 10.1155/2014/580385

**Published:** 2014-01-22

**Authors:** Jubayer Jalil, Mamun Bin Ibne Reaz, Mohammad Arif Sobhan Bhuiyan, Labonnah Farzana Rahman, Tae Gyu Chang

**Affiliations:** ^1^Department of Electrical, Electronic and Systems Engineering, Universiti Kebangsaan Malaysia, 43600 UKM, Bangi, Selangor, Malaysia; ^2^School of Electrical and Electronics Engineering, Chung-Ang University, Seoul 156-756, Republic of Korea

## Abstract

In radio frequency identification (RFID) systems, performance degradation of phase locked loops (PLLs) mainly occurs due to high phase noise of voltage-controlled oscillators (VCOs). This paper proposes a low power, low phase noise ring-VCO developed for 2.42 GHz operated active RFID transponders compatible with IEEE 802.11 b/g, Bluetooth, and Zigbee protocols. For ease of integration and implementation of the module in tiny die area, a novel pseudodifferential delay cell based 3-stage ring oscillator has been introduced to fabricate the ring-VCO. In CMOS technology, 0.18 *μ*m process is adopted for designing the circuit with 1.5 V power supply. The postlayout simulated results show that the proposed oscillator works in the tuning range of 0.5–2.54 GHz and dissipates 2.47 mW of power. It exhibits a phase noise of −126.62 dBc/Hz at 25 MHz offset from 2.42 GHz carrier frequency.

## 1. Introduction 

The operating frequency ranges of current RFID systems established for international standards extend from 135 kHz to 2.45 GHz in applications of biomedical, supply chain, public transport, and many more areas [1, 2]. Despite its emergence in today's world, RFID deployment in numerous applications is a key challenge for technologist due to multiple standardization issues and expensive vendor-specific readers. Moreover, RFID transponders (also known as tags) operated in several bands—high frequency (HF) (13.56 MHz), ultra-high frequency (UHF) (860–915 MHz), and microwave band (2.4 GHz)—have limited operational range to cover less than 2 m to maximum 9 m [3]. To overcome these shortcomings, the concept of readerless RFID system based on IEEE 802.11 b (Wi-Fi technology) and IEEE 802.15.4 (Zigbee) compliant standards has been proposed in [4, 5], respectively. In these systems, implemented RFID transponders are battery-powered active devices and their operating frequency is 2.4 GHz (unlicensed ISM band). Effective use of the active transponder's power is undoubtedly a crucial concern to implement in these RFID systems.

Analog transceiver in gigahertz range RFID transponder dissipates substantial amount of power during communication. In the RF transceiver, one of the key blocks is the frequency synthesizer or local oscillator. The phase locked loop (PLL) based frequency synthesizer is very popular in RF application from the outset. A PLL is a combination of phase detector (PD), low pass filter (LPF), voltage-controlled oscillator (VCO), and frequency divider. In a PLL, the most power hungry module is VCO which generates frequency and changes the oscillating frequency by varying control voltage. Nowadays, high frequency VCOs are built on complementary metal oxide semiconductor (CMOS), BiCMOS, SiGe, InP, and GaAs technologies for various ranges of frequencies. In comparison with other technologies, CMOS dominates the semiconductor industry nearly three decades due to its rapid evolution, continual downscaling of process, lower power dissipation, and reduced cost of fabrication [6–9]. Until now, LC-type and RC-type of CMOS VCOs have been used in wireless communication systems [10]. Typically, a VCO performance is analyzed by low phase noise, low power consumption, low voltage operation, high-speed oscillation, multiphase application, supply sensitivity reduction, simplified integration method, small layout area, and wide tuning range. So far, LC-based VCO has low level of phase noise among all VCOs. However, it has narrow tuning range, greater power consumption, and large die area [11]. In addition, it is very difficult to integrate inductor in digital CMOS technology. These limitations on LC-VCO can easily be overcome by ring-VCO.

This research work focuses on designing a low power pseudodifferential (PD) delay cell based ring-VCO with improved phase noise performance, which is suitable for high data rate active RFID transponder compatible with Wi-Fi, Bluetooth, and Zigbee networks. The architecture and operation of the proposed VCO will be presented in Section 2. The designing of the PD delay cell of the VCO will be described in Section 3. Finally, the postlayout simulation results will be discussed and compared in Section 4, followed by conclusion.

## 2. Three-Stage Ring-VCO Architecture

In general, a number of delay cells, which are connected in a positive or regenerative feedback loop for building a basic ring oscillator (RO), are the main basis of ring-VCO. Unlike LC, the on-chip RO is an inductor-free circuit and it is built by delay stages without a frequency selective network (resonant circuit). These delay stages or delay cells are inverting amplifiers. A common practice of ring-VCO implementation in CMOS process is accomplished by either single-ended or differential topology of delay cell. The single-ended ring topology comprises inverters and each inverter is made up of an NMOS and PMOS transistors. On the other hand, a differential topology includes a load (active or passive) with an NMOS differential pair. Currently, differential circuit topology is getting acceptance among designers as it has common-mode rejection of supply and substrate noise [17]. Moreover, it could be formed by odd or even number of stages and is possible to achieve both in-phase and quadrature outputs in DROs [18].

Choosing optimum number of stages for construction of high frequency oscillator is an important part of designing ring-VCO. Two, three, and four stages are common structures for the development of DRO in wireless communication systems. Several novel delay cells have been demonstrated to compose the two-stage ring-VCO, but extra power is inevitably needed to provide an excess phase shift for oscillation satisfying Barkhausen criterion. On the other hand, implementation of 4 stages of RO consumes considerable amount of power due to additional stages. Though 3-stage ring oscillator cannot produce quadrature outputs like 2-stage or 4-stage RO, it is faster than its 4-stage counterpart. Moreover, in 3-stage RO, fulfillment of proper start-up conditions can easily be attained unlike even number ROs, where latch-up frequently occurs. Thus, for designing the proposed VCO, the 3-stage RO is chosen to increase the oscillation and to reduce power consumption concurrently.

**Figure 1:**
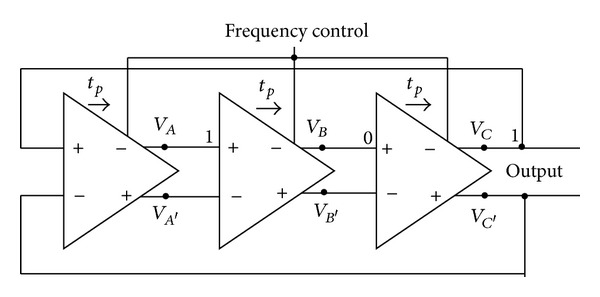
Block diagram of the 3-stage ring-VCO.

**Figure 2:**
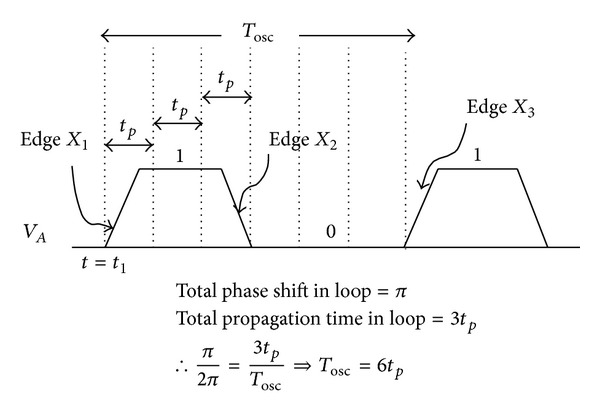
Corresponding waveform of the 3-stage ring-VCO and total period calculation.

For incorporation of 3-stage, single delay loop ring oscillator, only three of differential amplifiers are connected in a single delay path formation as shown in Figure 1. Dual delay loop, a technique for achieving maximum frequency levels, is not considered due to additional transistors and power consumption. Principle operation of the proposed oscillator structure is that if one of the nodes is excited, the pulse propagates through all the stages and reverses the polarity of the initially excited node. To explain the basic working principle of the DRO, let us consider a three-stage DRO (*N* = 3), in which at time *t*_1_, the output of the first stage, voltage changes to logic 1 (denoted by edge *X*_1_) as shown in signal waveform in Figure 2. When this logic 1 propagates to the end, it creates a logic 1 at the third stage, which, when fed back to the input of the first stage, creates a logic 0 in the first stage output denoting edge *X*_2_. When this logic 0 is propagated again through the loop, it toggles the output voltage of the first stage and trigger edge *X*_3_. For every single cycle, there are a downward and an upward transition, and the intrinsic propagation delay times of each delay cell, high-to-low (*t*_PHL_) and low-to-high (*t*_HPL_), are associated with these transitions. Nevertheless, *t*_PHL_ and *t*_HPL_ could be equal or not depending on the specific delay cell configurations, and so the average propagation delay can be implied by the arithmetic mean of transition times, (*t*_PHL_ + *t*_HPL_)/2.

For start-up and oscillation criteria, the transfer function for this ring oscillator with the number of stages (*N*) set to three can be represented as

(1) $$\begin{array}{c} H\left(S\right)=\frac{-A_{0}^{3}}{{\left(1+\left(S/\omega_{0}\right)\right)}^{3}}, \end{array}$$

where *A*_0_ denotes voltage gain of each delay cell and *ω*_0_ denotes 3 dB bandwidth at each stage.

One of the criteria for oscillation is the phase shift of 180°; that is, each stage contributes with 60° of phase shift for three-stage RO, and the frequency at which it occurs is given as

(2) $$\begin{array}{c} \omega_{\text{osc}}=\omega_{0}\tan\left(\frac{180^{{}^{\circ}}}{3}\right). \end{array}$$

The other criterion for oscillation is the loop gain (at *ω*_osc_) which must be greater than 1 to achieve the minimum voltage gain per stage. Consider

(3) $$\begin{array}{c} 1=\frac{A_{0}^{3}}{{\left[\sqrt{1+{\left(\omega_{\text{osc}}/\omega_{0}\right)}^{2}}\right]}^{3}}. \end{array}$$

By inserting the oscillation frequency expression of (2) into the gain equation (3), we can calculate the minimum voltage gain per delay cell:

(4) $$\begin{array}{c} A_{0}=2. \end{array}$$

For every signal cycle, there is a downward as well as an upward transition. Since the high-to-low (*t*_PHL_) and low-to-high (*t*_PLH_) propagation delays associated with these transitions are not usually equal, the average propagation delay is given by

(5) $$\begin{array}{c} T=\frac{\left(t_{\text{PHL}}+t_{\text{PLH}}\right)}{2}. \end{array}$$

A propagating signal has to pass twice through the chain of delay cells, for a total delay of 2*NT*, to complete one period. The oscillation frequency for an *N*-stage ring is derived from the average propagation delay (*T*) of the inverter. The frequency of the oscillation (*f*_osc_) is expressed as

(6) $$\begin{array}{c} f_{\text{osc}}=\frac{1}{2NT}. \end{array}$$

## 3. Design of Proposed Delay Cell Architecture 

**Figure 3:**
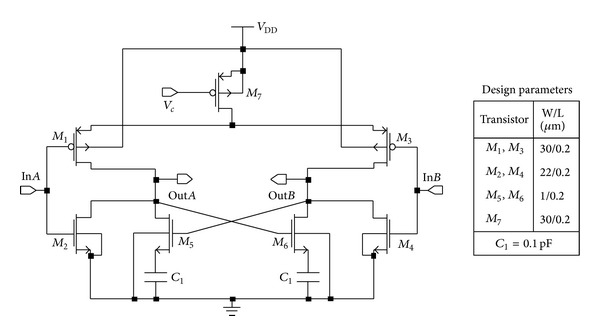
Schematic diagram of the proposed delay cell.

In this research, a pseudodifferential (PD) configured delay cell architecture for the ring-VCO has been introduced as shown in Figure 3. Due to the tail current source in true differential amplifier, the common-mode gain is reduced by increasing the output resistance of the bias current source. Conversely, the absence of tail current source in PD amplifier results in a large common-mode gain [19]. Moreover, since PD cell alleviates necessity of tail current transistor, it is free from flicker noise [11]. Additionally, it avoids redundant bias circuit which occupies a large space in integrated circuit (IC).

According to Figure 3, a pair of CMOS differential push-pull inverter is used as inputs in the new delay cell architecture. This input pair can be stated as complementary input pair. Each complementary input consists of two different sizing of PMOS and NMOS transistors. Additionally, two cross-coupled NMOS transistors are connected in parallel with input NMOS transistors. These cross-coupled NMOS transistors are introduced for fast switching speed. Sizes of all four NMOS in the cell are chosen unequally as well. In addition, a serially connected load capacitor with cross-coupled NMOS is employed in parallel with each NMOS input. Here, frequency tuning is achieved through a PMOS transistor connected directly with supply voltage.

The operation of the delay cell can be illustrated considering half-cell circuit. According to Figure 3, while the input In*A* becomes high (near VDD), the input In*B* becomes low (equal to zero volt). This occurrence turns on NMOS (*M*_2_) of the node In*A*. In addition, cross-coupled NMOS (*M*_5_) is turned on due to close of PMOS (*M*_3_) at the input node In*B*. On the other hand, PMOS (*M*_1_) of the input node In*A* remains off. Then, voltage of the output node Out*A* is grounded. During that period, charge from the capacitor (*C*_*l*_), which is serially connected with *M*_5_, is discharged; or in other words, a path is formed, which sinks current from Out*A* to bring its potential to 0 V. Similarly, if the input In*A* turns into 0 (zero) V, then the input In*B* becomes high (near VDD). Now, zero potential of the input In*A* turns on PMOS (*M*_1_) and turns off NMOS (*M*_2_), simultaneously. A cross-coupled NMOS (*M*_5_), connected in parallel with the input NMOS (*M*_2_), remains switched on till the complete discharge of capacitor (*C*_*l*_) of the other half circuit of this cell because potential of the output node Out*A* becomes near VDD and turns on NMSO (*M*_6_). Now, the previously discharged capacitor (*C*_*l*_) recharges again through *M*_5_. Here, a PMOS tuning transistor (*M*_7_) controls both charging and discharging operations of the load capacitor and eventually the frequency of the oscillator varies.

**Figure 4:**
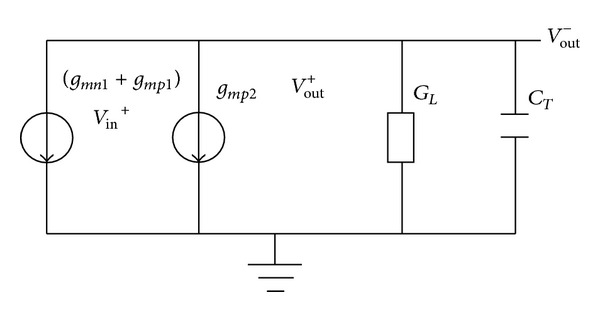
Small-signal equivalent circuit of the half circuit of the delay cell for frequency analysis.

To calculate the operating frequency of the ring oscillator, a half circuit of the proposed delay cell in Figure 4 is considered. The transfer function of the delay cell *A*_*v*_(*s*) is shown as follows:

(7) Av(s)=VoutVin=gmn1+gmp1GL−gmp2+sCT=(gmn1+gmp1)/(GL−gmp2)1+sCT/(GL−gmp2),

where *g*_*m*_ is the transconductance of the transistor, *C*_*T*_ is the total capacitance at the output node and *G*_*L*_ is the resistance load due to channel length modulation. Calculation of the operating frequency is derived as follows:

(8) A0=gmn1+gmp1GL−gmp2=2tan−1[ωosc(GL−g mp2 )/CT]=60°fosc=32π(GL−gmp2)CT=34π(gmn1+gmp1)CT.

## 4. Results and Comparisons

**Figure 5:**
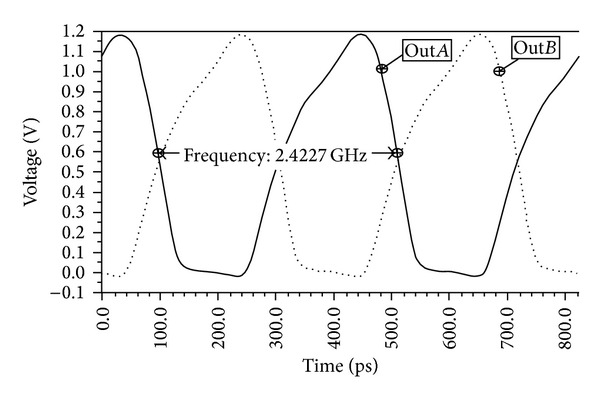
Simulated output of the proposed ring-VCO.

The proposed delay cell circuit has been verified by using the EldoRF simulator (Mentor Graphics). The process parameters for the transistors used in this work correspond to Collaborative Micro-electronic Design Excellence Centre (CEDEC) 0.18 *μ*m standard 1P6 M CMOS technology. To determine the operating frequency of the proposed delay cell circuit, the postlayout simulated output frequency of the ring-VCO is shown in Figure 5. Frequency of 2.42 GHz is achieved, while the control voltage is set to 0.1 V. To obtain this result, the supply voltage is set to 1.5 V and 0.1 pF of each load capacitor selected in the circuit. The proposed circuit arrangement and different sizes of the transistors make it possible to get the required frequency. The operating temperature of the circuit is set to 27°C.

**Figure 6:**
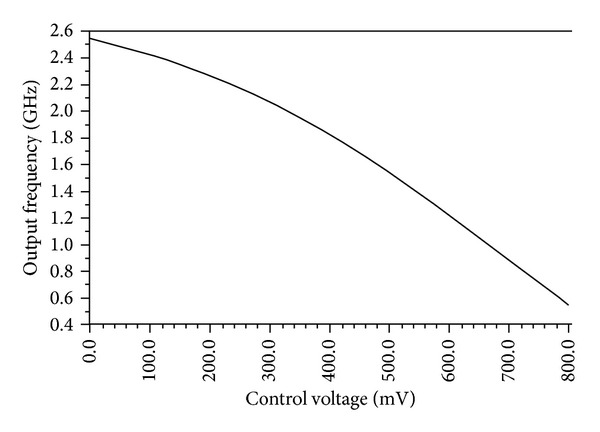
Tuning range of the proposed ring-VCO at 27°C.

In order to validate the proposed circuit in wide frequency range, the simulation is done at different control voltages. A frequency tuning range of 80% is attained from 0.5 GHz to 2.54 GHz applying 0.8 V to 0 (zero) V as shown in Figure 6. In the proposed architecture, it is observed in Figure 6 that a linear relationship has been established between control voltage and frequency of oscillation.

**Figure 7:**
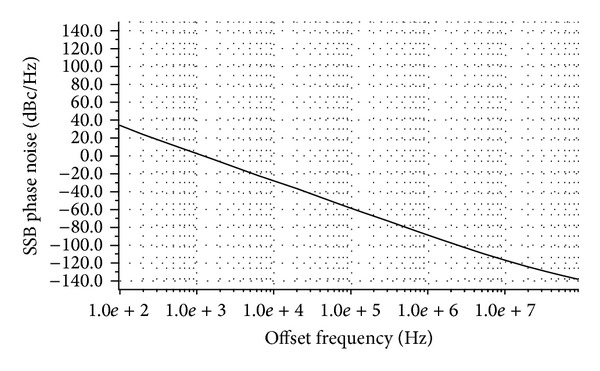
Single side-band (SSB) phase noise (PN) of the proposed ring-VCO.

Since IEEE 802.11 b protocol is required to generate frequency from 2.412 GHz to 2.484 GHz, the proposed delay circuit makes the ring-VCO working on this frequency range, which is certainly a key parameter of readerless, active RFID transponder. All major short range as well as long range communication standards, for example, GSM, DCS-1800, WLAN IEEE 802.11 b/g, IEEE 802.11 FH (Bluetooth), and Zigbee, operate in the frequency range of 0.8 GHz to 2.5 GHz, where the frequency range demands exceptional VCO performance. However, the VCO phase noise requirement of Wi-Fi is not relaxed than Bluetooth or Zigbee standards, rather stringent, as bit error rate (BER) of Wi-Fi is much higher. For IEEE 802.11 b protocol, keeping BER better than 10^−5^, the phase noise must be met to −126 dBc/Hz at 25 MHz offset [20]. In our design, we have achieved single side-band phase noise of −126.62 dBc/Hz at 25 MHz offset from the carrier 2.42 GHz as shown in Figure 7. To generate 2.42 GHz frequency, 2.47 mW of power has dissipated this oscillator.

The figure of merit (FOM) of the proposed ring-VCO can be calculated from the power dissipation and the phase noise of the simulated oscillation frequency by

**Figure 8:**
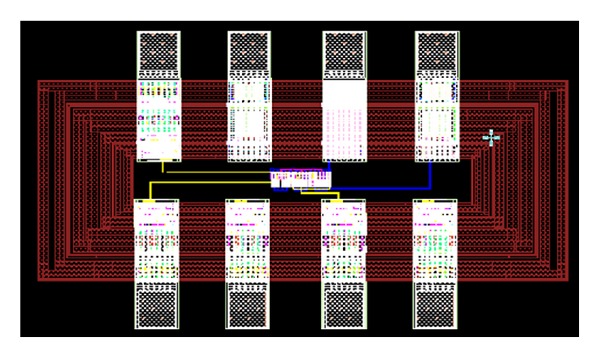
Layout of the pseudodifferential ring-VCO.

(9) FOMdB=L(Δω)+10log⁡(PowerDC/1 mW) −20log⁡(ω0/Δω),

where *L*(Δ*ω*) is phase noise in offset frequency and *ω*_0_ is the frequency of oscillation of the ring-VCO. The achievable FOM is found to be −162.4 dBc/Hz. A layout of the chip is shown in Figure 8, where the VCO core occupies an area (without PADs) of 145 × 64 *μ*m^2^.

The principle of industry oriented EDA tools (such as Mentor Graphics, Cadence, etc.) is expected to have the closest simulation result to the experimental result. Here, we have used Mentor Graphics to design, simulate, and draw layout of our proposed design of VCO. Therefore, the postlayout simulation will be expected to agree with the actual measurement result after IC fabrication. The postlayout design (Figure 8) has been sent for fabrication using standard 0.18 *μ*m CMOS process including PADs and buffer circuit.

Table 1 summarizes the performance of our proposed ring-VCO along with other research works' results of ring-VCO for comparison. Compared to [15], this ring-VCO has better phase noise and high value of FOM at the expense of modest power. Its power consumption is significantly lower than [12–14, 16]; and finally, its wide tuning range is notable than other reported works except that of [13].

## 5. Conclusion

Despite the continuous improvement in the state of the art of VCOs in downscaling CMOS process, they still remain the most crucial blocks for high frequency PLLs. In this paper, a ring-VCO has been introduced for active, readerless RFID transponder compliant with established short-range communication networks, such as Wi-Fi, Bluetooth, and Zigbee. This proposed ring oscillator-based VCO is achieved by employing 3-stage delay cell, where each delay cell is configured as pseudodifferential circuit with complementary push-pull input. Wide tuning range and phase noise performance of the oscillator have been evaluated through postlayout simulation results. The comparison results show that the proposed ring-VCO's comparable performances offer the benefit of operating in a low-voltage environment, reduced power dissipation, and tiny layout area. The proposed oscillator's better phase noise performance is sufficient to achieve maximum data rate of 11 Mbps for Wi-Fi transceivers.

## Figures and Tables

**Table 1:** Performance comparisons of CMOS ring-VCO.

Architecture	Operating frequency (GHz)	Tuning range (GHz)	Phase noise (dBc/Hz)	Offset (MHz)	Supply voltage (V)	Power (mW)	FOM (dBc/Hz)	CMOS process (µm)	Published year, [Ref.]
2-stage, single delay loop	0.85	0.186–1.5	−113.5	0.6	1.8	11.38	−165.96	0.18	2008, [12]
3-stage, dual delay loop	4.09	0.479–4.09	−94.08	1	1	10	−156.28	0.18	2011, [13]
4-stage, dual delay loop	—	1.77–1.92	−123.4	10	1.8	13	—	0.18	2011, [14]
3-stage, single delay loop	2.4	2.34–3.11 (24.75%)	−113	10	1.05	2	−157.6	0.13	2011, [15]
3-stage, single delay loop	0.866	0.381–1.15	−126	10	3.3	7.48	−156	0.35	2012, [16]
3-stage, single delay loop	2.42	0.5–2.54 (80%)	−126.4 −118	25 10	1.5	2.47	−162.4 −161.74	0.18	Proposed work
